# Physical Health Views Among Individuals Experiencing Mental Illness: A Mixed‐Methods Study of Self‐Reported Health and Contributing Factors

**DOI:** 10.1111/inm.13489

**Published:** 2025-01-27

**Authors:** Stefan Sebastian Heinz, Anthony John O'Brien, Matthew Parsons, Cameron Walker

**Affiliations:** ^1^ University of Waikato Hamilton New Zealand; ^2^ University of Auckland Auckland New Zealand

**Keywords:** barriers, holistic care, mental health, motivation, nursing, physical health, psychiatric, SF12

## Abstract

Severe mental illness is linked to poor physical health and shorter life expectancy, yet research on how individuals experiencing mental illness view and on improve their physical health is limited. This study investigates the perceptions of individuals experiencing mental illness regarding their physical health, utilising a mixed‐methods approach. Phase I involved quantitative and qualitative data from an online Qualtrics survey, which included the 12‐item Short Form (SF‐12) survey to measure participants' quality of life and assess self‐reported physical and mental health. Key findings from Phase I revealed significant relationships between lower Physical Component Summary (PCS) scores and factors such as the frequency of GP visits. Additionally, exercise preferences were found to significantly impact Mental Component Summary (MCS) scores, with individuals who preferred a mix of exercise settings reporting higher MCS scores compared to those who exercised alone or with a training partner. Phase II explored these findings further through semi‐structured interviews, where participants discussed themes including physical health perceptions, the role of medication and the importance of the general practitioner relationship. Thematic analysis revealed five main barriers to improving physical health: accessibility and availability of services, motivation, staff attitudes, medication side effects and the experience of diagnostic overshadowing. Participants reported viewing physical and mental health as interconnected and expressed a desire for more collaborative care. The results suggest that strengthening the relationship with GPs and increasing awareness of medication side effects may improve physical health outcomes for individuals experiencing mental illness. Mental health nurses can play a pivotal role in enhancing physical health outcomes by monitoring, supporting health‐improving strategies and facilitating access to primary care services.

## 1 Introduction

Health is a complex and multifaceted concept encompassing physical, mental, social and spiritual dimensions. The World Health Organisation (WHO) defines health as ‘a complete physical, mental and social well‐being and not merely the absence of disease or infirmity’ (2022, para 2). This definition recognises the importance of holistic health, where all aspects of health are interconnected and must be balanced for overall well‐being. Indigenous New Zealand (NZ) health models, such as the Māori model, Te Whare Tapa Whā, also emphasise the interconnectedness of physical, mental, social and spiritual health (Durie 1985). This model recognises that each aspect of health must be balanced to achieve optimal health and well‐being.

Physical health is a critical issue for individuals experiencing mental illness. Mental health, especially severe mental illness, is disproportionately linked to physical illnesses such as coronary heart disease (Cunningham et al. 2014), hypertension (Wells et al. 2018) and diabetes (Robinson et al. 2018). In fact, the life expectancy for individuals experiencing serious mental illness is 18 years shorter than those without (Plana‐Ripoll et al. 2019). These physical health challenges are often exacerbated by the stigma and discrimination associated with mental illness, leading to inadequate access to healthcare services and reduced quality of care (Knaak, Mantler, and Szeto 2017). In addition, diagnostic overshadowing and a silo mentality in primary and secondary healthcare services contribute to poor health outcomes in people with complex mental and physical health needs (Cole and Padmanabhan 2012; Sinding et al. 2013). Mental health nurses are perceived as key when addressing these complex health needs; however, mental health nurses do not always see physical health care as part of their role (Happell et al. 2012) and may lose opportunities to improve the physical health of individuals experiencing mental illness.

Māori, the indigenous people of NZ, experience inequitable healthcare delivery and health outcomes (Ministry of Health 2020). For example, Māori have significantly lower cancer survival rates than non‐Māori (Obertová et al. 2015). This inequity is influenced by various factors, including socioeconomic status, cultural barriers and multigenerational trauma caused by colonisation (Smallwood et al. 2021). These disparities highlight the urgent need for culturally appropriate and accessible healthcare services that prioritise the unique health needs and experiences of Māori. Furthermore, the Crown has obligations under Te Tiriti o Waitangi (1840) to uphold Māori health equity, addressing the ongoing impacts of colonisation. Recognising these disparities, the Ministry of Health has implemented initiatives to promote equitable healthcare access for Māori (Curtis et al. 2022; Ministry of Health 2023).

Mental health nursing is crucial in addressing the interconnectedness of physical and mental health, particularly for marginalised and indigenous communities. Mental health nurses are uniquely qualified to provide comprehensive care that addresses the physical health needs of individuals experiencing mental illness, including those from marginalised and indigenous populations. They advocate for high‐quality healthcare treatments, such as routine screenings, health assessments and physical health interventions (Wynaden et al. 2016). Mental health nurses combat stigma and discrimination related to mental illness, ensuring access to treatments and bridging healthcare disparities (Bates and Stickley 2013). They provide culturally appropriate care and advocate for integrated care that considers both physical and mental health needs, breaking down silos between primary and secondary care (Duggan et al. 2020).

Mental health nurses play a crucial role in health promotion and education initiatives, empowering individuals experiencing mental illness to manage their physical health and make informed decisions about their well‐being (Hennessy and Cocoman 2018). They provide information on healthy lifestyles, support access to resources and promote self‐care practices that address both physical and mental health (Gray and Brown 2017). Mental health nurses improve health outcomes for marginalised and indigenous populations by delivering holistic, culturally appropriate and integrated care.

## 2 Background

Numerous studies have explored approaches and strategies to manage and improve the physical health of individuals experiencing mental illness. However, these studies have focused on strategies shaped by health professionals. This approach is insufficient for individuals experiencing mental illness with individualised needs and strengths and should be supplemented with research their perceptions. An existing NZ study investigated how individuals experiencing mental illness perceived their physical well‐being in inpatient units in Christchurch (Every‐Palmer et al. 2018). However, was limited to participants whose primary diagnosis was schizophrenia.

### 2.1 Aim

Given the lack of research regarding the physical health perception of individuals experiencing mental illness, this study aimed to identify and evaluate their views and identify strategies for improvement in primary and secondary health services. The following four research questions aim to explore the physical health of individuals experiencing mental illness:

1. How do individuals experiencing mental illness perceive their physical health needs?
2. What barriers and supports influence physical health in individual experiencing mental illness?
3. What are key factors affecting physical health for those experiencing mental illness?
4. How do individuals experiencing mental illness view the role of healthcare in their physical health?

## 3 Methods

### 3.1 Design

The following main research problem was addressed using an explanatory sequential mixed‐method methodology (Creswell 2011): (1) measured variables affecting individuals experiencing mental illness perception of their physical health, and (2) qualitatively explored individuals experiencing mental illness perception of their physical health and their relationship with their general practitioner.

Survey and interview questions were co‐designed with service user advisors, cultural advisors and peer reviewed by a sample of individuals utilising mental health services (*n* = 8). Following this review, less medical language was used. The first phase utilised a Qualtrics online survey with 32 questions, comprising the SF‐12, general health‐related (e.g., physical activity preferences and interests, desire for health improvement, GP access, and cultural and support needs) and demographic questions. The SF‐12, developed by Ware, Kosinski, and Keller (1996), is a widely used instrument that assesses health‐related quality of life. It measures physical and mental health across eight dimensions, including physical functioning, role limitations because of physical and emotional problems, bodily pain, general health perceptions, vitality, social functioning and mental health.

The SF‐12 generates two summary scores: the PCS and the MCS. These scores are based on weighted algorithms that normalise data to a population mean of 50 and a standard deviation of 10 for population comparison (Turner‐Bowker and Hogue 2014). Higher scores indicate better health outcomes. The SF‐12 has been tested in populations including NZ samples and has shown has demonstrated strong validity and reliability (Frieling, Davis, and Chiang 2013). Although there are no universal strict cutoff scores for the SF‐12, lower scores can reflect reduced health‐related quality of life and can be clinically meaningful for detecting people with high level of health concerns. Statistically significant responses from Phase I were explored in Phase II. In the second phase, the researcher employed a culturally sensitive interview guide depending on preference, which encompassed both an opening and closing prayer (karakia) and seamlessly integrated a process of establishing relationships (whakawhanaungatanga) to enrich the depth and cultural relevance of the conversations. The interviews lasted about an hour.

### 3.2 Inclusion and Exclusion Criteria

Mental health service administrators distributed online surveys to individuals enrolled with mental health services. According to the exclusion criteria, all participants were selected from the government‐funded healthcare organisation Te Whatu Ora located within a geographic footprint of the Waikato District in NZ. Excluded were participants aged 17 and below, those accessing forensic inpatient services (due to lack of access to mobile phones), and individuals in acute inpatient services, or had only a landline recorded on their file. This focus on mobile phones was essential, as the survey link was distributed via mobile numbers, ensuring accessibility for participants.

### 3.3 Data Collection

Data collection occurred between in 2021, with a mental health service administrator sending an e‐text to the mobile numbers of Waikato DHB mental health service users. The mobile numbers were provided to the administrator by the Waikato DHB's data analyst according to the exclusion and inclusion criteria. A total of *N* = 2081 e‐texts were distributed to individuals enrolled with Adult Mental Health Services via the Waikato DHB's e‐text system, inviting service users to participate in the survey. Interested participants were asked to open the Qualtrics hyperlink to complete the survey.

In the final Qualtrics survey question, participants interested in participating in the second part of the study were prompted to leave their mobile number or email address. These contact details were extracted separately from the gathered data on Qualtrics. Subsequently, the researcher contacted these participants via telephone and sought their verbal consent to participate in the interviews. During this contact, the researcher explained the conduct and nature of the research study, including anonymity, the right to withdraw and provided additional information such as the interview timeframe. Participants were encouraged to bring a support person to the interview. The participants' details were also checked to confirm that they met the study's inclusion criteria.

The first four Māori and the first four non‐Māori who consented to participate were selected for the semi‐structured interviews. Interviews were held in mental health service facilities. Ethics committee approval was obtained for this study to ensure compliance with ethical standards when collecting data from individuals.

### 3.4 Data Analysis

Quantitative data collected from the survey were extracted to SPSS version 28.0.1.0 and analysed using descriptive statistics. Initial univariate analyses were conducted to identify candidates for inclusion in the model, focusing on significant relationships between individual independent variables and the dependent variable. Following this, backward regression was performed, systematically removing the least significant predictors until only those with a statistically meaningful impact remained. Finally, 95% confidence intervals for the coefficients were generated to assess the precision and reliability of the estimates. Free text responses to the survey and the interview findings were analysed in an inductive approach, with transcripts coded in parallel by the authors and categorised into subthemes and themes using the NVivo software.

## 4 Phase I—Quantitative Findings

In Table 1 percentages may not total 100% due to non‐responses or missing data. Participants were not required to answer every question, resulting in incomplete data points for some items. Out of the 2081 invitations, 167 (8%) responded to the survey. Although the overall response rate was low, the demographic breakdown in Table 1 offers valuable insights into the representation of key groups.

**TABLE 1:** Study sample.

Descriptors	*n* = 167	100 (%)
Sex		
Female	90	53.9
Male	40	24
Gender diverse	6	3.6
Age		
18–24 years old	28	16.8
25–34 years old	36	21.6
35–44 years old	24	14.4
45–54 years old	29	17.4
55–65 years old	13	7.8
65+ years old	6	3.6
Mean age		
Mean years (SD)	38.26 (13.67)	13.7
Ethnicity		
Māori	50	29.9
Pasifika	4	2.4
Non‐Māori	82	49.1
Living		
Urban	104	59.9
Rural	17	10.2
Independent living	50	29.9
Living at home with whanau	49	29.3
Flatting with others	24	14.8
Supported accommodation	5	3.0
Emergency accommodation	5	3.0
Time with mental health services		
< 1 year	36	21.6
1–5 years	40	24.0
6–10 years	20	12.0
11–20 years	27	16.2
> 20 years	9	5.4

### 4.1 GP Contact and Health Status

The majority of participants had a regular GP and saw their GP at least once a month (*n* = 29, 17.4%), once in a couple of months (*n* = 35, 21%), once every 3 months (*n* = 34, 20.4%), once every 6 months (*n* = 15, 9%), once every year (*n* = 14, 8.4%), and less than a year (*n* = 5, 3%). Participants were also asked if they had a physical health condition or disability that limits them and that has lasted 6 months or more. High blood pressure, asthma, high cholesterol and type 2 diabetes were the most identified physical health diagnoses. A total of 13.7% reported physical health conditions that limited their quality of life within the last 6 months or more, indicating that every sixth participant faces physical health conditions that limit their quality of life.

#### 4.1.1 Mental Health Service Contact and Mental Health Medication

Mental health service contact and use of mental health or addiction medication were found to be high among participants, with many receiving support from mental health and addiction services over varying durations: < 1 year (21.6%), 1–5 years (24%), 6–10 years (12%), 11–20 years (16.2%) and > 20 years (5.4%). A majority of participants, 116 (69.5%), reported taking regular medication for their mental health or addiction. However, only 46 (27.5%) of the individuals received information about potential physical side effects of their medication, whereas 36 (21.6%) did not receive such information.

### 4.2 Physical Health Improvements and Preferences

Physical health improvements and preferences were discussed, with over 82.1% of participants expressing some interest in improving their physical health. Regarding specific strategies to enhance their physical health, 65.3% preferred physical activity, whereas 60.5% selected healthy eating. Other strategies, such as smoking cessation (19.2%), reducing alcohol intake (15.6%) and substance use reduction (13.8%), were the less favoured. Many participants preferred physical activities such as walking (53.9%), yoga (35.3%) and swimming (34.7%). Of the respondents, 78 (46.7%) indicated a need for Kaupapa Māori support groups, whereas 17 (10.2%) reported no need for such support.

### 4.3 SF‐12 Analysis

The SF‐12 questionnaire was used to assess participants' functional well‐being with a mean PCS of 42.7 and a mean MCS of 33.2.

The univariate analysis revealed several significant relationships between the PCS and independent variables. For the question, ‘Were you told about potential physical side effects of your mental health or addiction medication?’ there was a significant association with PCS (*p* = 0.049). Participants who were informed about side effects had a mean PCS of 43.3 (95% CI: [41.5, 45.1]), compared to 39.4 (95% CI: [36.9, 42.0]) for those who were not informed and 40.1 (95% CI: [36.9, 43.3]) for those unsure. This suggests that being informed about side effects is associated with higher PCS scores. The variable ‘Do you have a physical health condition or disability that limits you, and that has lasted for 6 months or more?’ showed a highly significant relationship with PCS (*p* = 0.001). Those with a condition had a mean PCS of 40.1 (95% CI: [37.1, 43.0]), whereas those without had a mean PCS of 44.2 (95% CI: [42.4, 45.9]). This indicates that individuals with long‐term physical health conditions report lower PCS scores. The frequency of GP visits was also significantly associated with PCS (*p* = 0.005). The mean PCS scores were 37.9 (95% CI: [35.2, 40.6]) for those visiting at least once a month, 42.5 (95% CI: [41.1, 43.9]) for those visiting once in 2–6 months and 42.8 (95% CI: [40.0, 45.5]) for those visiting less than once every 6 months. This suggests that more frequent GP visits are associated with lower PCS scores. These findings highlight the importance of both physical health conditions and healthcare engagement in determining functional well‐being, as measured by PCS.

Following the univariate analysis, backward regression was employed to refine the model and identify the most significant predictors of PCS scores (Table 2). The final model retained one key independent variable: frequency of GP visits. The model indicated a strong relationship, showing that individuals who visit their GP more frequently tend to report higher physical health scores, highlighting the importance of regular healthcare access (Table 3).

**TABLE 2:** Regression analysis for PCS and MCS.

Model		Unstandardized coefficients		Standardised coefficients	*t*	Sig.	95.0% confidence interval for *B*	
		*B*	SE	Beta			Lower bound	Upper bound
3	(Constant)	36.834	1.975		18.654	< 0.001	32.927	40.741
	How often do you usually see your GP?	2.375	0.950	0.216	2.501	0.014*	0.496	4.254
	a. Dependent variable: PCS							
2	(Constant)	32.948	1.120		29.409	< 0.001	30.731	35.164
	Do you like to do things on your own or to be part of a group‐based activity?	1.171	0.486	0.207	2.407	0.018*	0.208	2.133
	a. Dependent variables: MCS							

* *p* ≤ 0.05.

**TABLE 3:** PCS—How often do you usually see your GP? and mental health and addiction medication.

How often do you usually see your GP?	PCS
At least once a month	37.9
Once in 2–6 months	42.5
Less than once every 6 months	41.6
Do you like to do things on your own or to be part of a group‐based activity?	MCS
Exercising alone	34.1
Exercising with a training buddy	35.7
I enjoy all of these, and would like a mixture of them	38.0

The univariate analysis revealed several significant associations with MCS scores. Preferences for exercise showed a significant relationship with MCS (*p* = 0.013). Those who exercised alone had a mean MCS score of 34.1 (95% CI: [32.7, 35.5]), whereas those who exercised with a training buddy had a mean score of 35.7 (95% CI: [32.7, 38.7]), and those who preferred a mix of both had a higher mean score of 38.0 (95% CI: [35.2, 40.8]). This suggests that enjoying a variety of exercise settings is associated with higher MCS scores. District of residence was also significantly related to MCS (*p* = 0.04). The mean MCS scores for the main districts were as follows: Hamilton (35.2, 95% CI: [33.7, 36.7]), Hauraki (29.0, 95% CI: [13.1, 44.9]), Waikato (40.1, 95% CI: [34.2, 46.1]), Matamata‐Piako (35.2, 95% CI: [31.6, 38.7]) and South Waikato (33.9, 95% CI: [29.3, 38.4]). Because of a low response rate, other districts were excluded from the analysis.

After conducting the univariate analysis, backward regression was used to refine the model and identify the most significant predictors of MCS scores (Table 2). The final model included one key variable: exercise preferences. Participants who indicated a preference for group‐based activities reported higher MCS scores, suggesting that social engagement positively correlates with mental health outcomes (Table 3). Other variables in this model did not reach statistical significance, indicating that the preferences for activities were the most notable predictors of mental health scores.

### 4.4 Free‐Text Response to Open‐Ended Questions

In the Qualtrics survey, 43% (*n* = 72) of participants responded to the question, ‘how do you think physical health care could be improved for people with mental illness or addiction issues?’ and a total of 18% (*n* = 30) participants responded to the ‘tell us more’ option as part of the question, ‘were you told about potential physical side effects of your mental health or addiction medication?’. The themes arising from the analysis were: accessibility and availability, people wanting to be healthy, motivation, staff attitude and medication.

Accessibility and availability were significant concerns, with many participants reporting a preference for free or subsidised costs to access gym memberships and tailored programs. However, barriers to accessing mental health services, such as GP costs and long waiting times for doctor appointments or rehabilitation centres, were also identified.

People wanting to be healthy captures perceptions of the benefits of a healthier lifestyle, including exercise, healthy eating and a drug‐free approach. Some participants suggested having a support person or role model to boost their willingness to be physically active. Inequitable access to mental health care in primary services posed further obstacles. Participants' desire for gradual independence and increased agency drove them to adopt healthy and sustainable behaviours, gravitating towards good health. Most respondents felt that physical exercise positively impacted their mental health, with some experiencing a relief from stressful thinking during exercise.

Motivation was a barrier to lifestyle changes, influenced by sedating medication or mental illness symptoms. Self‐efficacy played a significant role, impacting individuals' adaptation to health‐promoting behaviours. The survey underscored the importance of support and encouragement to help people with mental health issues overcome barriers and improve their physical health. Participants identified discrimination, stigmatisation and negative attitudes towards people with mental illness as barriers to delivering appropriate care.

Staff attitudes were a significant issue, with many participants feeling unheard and doctors not taking their concerns seriously. Listening to mental health service users' concerns is vital for their care and recovery, necessitating health professionals to treat their concerns seriously. *Stigma and racism* were identified as barriers to improving physical health. Mental health service users faced limitations because of being considered high‐risk, leading to restricted options and feelings of debilitation. The study highlighted the importance of focusing positively on other aspects of one's life and limitations.

Medication played a significant role in participants' physical health perceptions and experiences. Thematic analysis identified three subthemes: negative experience, side effects and taking initiative. Negative experiences stemmed from a lack of information provided by psychiatrists about potential side effects, impacting long‐term development. Some participants received no support for side effects noticed months or years after starting medication. Side effects include weight gain, reduced energy, tiredness, dry mouth, increased smoking urge, sleep difficulties and heart palpitations. Taking the initiative to understand prescribed medications is crucial for mental health and side effects of mental health and addiction medication. Participants often research or consult their psychiatrist about medication, but it can be challenging, especially for those with low energy levels due to psychosis, depressive episodes, or sedating medications. Clinicians' perceptions of non‐adherence, psychosis and depressive episodes may also hinder taking the initiative.

## 5 Phase II—Interview Findings

Although all participants were engaging with mental health services and the participant group was of heterogeneous in terms of age, gender, ethnicity and diagnosis and were using different mental and physical health services. The findings from the interviews revealed three themes, including physical health perception, the role of medication and the importance of the relationship with the GP.

### 5.1 Physical Health Perceptions

The analysis highlighted that participants had different views concerning the concept of physical health, their physical perception and physical health strategies. This section not only pinpoints differences in physical health perceptions but also identifies similarities within the heterogeneous participant group. Regarding health, responses saw physical health as part of a biomedical, systemic, functional, or holistic health concept.

Although one participant referred to physical health through a biomedical lens as ‘healthy [P4]’ and with ‘no major illnesses [P4]’, another participant [P5] had a systemic view of health. In their opinion, physical health includes ‘a lot of systems [P5]’ which are ‘functioning [P5]’ together. Furthermore, they explained that ‘when one system is not functioning properly in your body, there's a chain reaction that sets off other problems [P5]’. In addition to the systems view of physical health, participants also spoke about physical health in the context of a functional health concept, expressed as, for example, ‘the ability to do common tasks everyday things [P3]’.

Physical health was often described through a functional health lens. For example, responses emphasised physical health as wellness and functioning. In terms of physical health, ‘harmony’ was used to define the equilibrium of one's functioning and wellness. Although physical health was described as one's state of wellness in which the body functions in harmony, physical health was perceived as an element of an entire systemic construct which involves mental, social and spiritual health. One participant referred to physical health as an interconnected construct and described it through the lens of Durie's (1985) holistic health model Te Whare Tapa Whā.

Regardless of the different views on physical health, the link between physical and mental health was evident. In all interviews, participants spoke about the significance of physical health concerning their mental health. The following comments demonstrate this:

P5: Physical well‐being can't function on its own. It can't function without mental well‐being. Everything must function holistically and in harmony.

P4: I mean, the mind is directly related to physical health and vice versa.

Physical and mental health were either seen as a linear or parallel construct. Other responses stressed that ‘physical well‐being can't function on its own [P5]’, emphasising the parallel between physical and mental health. Although ‘physical symptoms can be exacerbated by mental health issues [P1]’, ‘physical issues can cause mental issues [P1]’. For example, self‐isolating behaviour due to physical pain contributes negatively to one participant's mental health. Concerns by other participants highlighted how mental illness contributes negatively to their physical health. Responses demonstrated that signs and symptoms of schizophrenia and depression led to avolition and self‐isolation and increasing weight. In some other cases, ‘stress’ was perceived as a crucial trigger for feeling ‘on edge [P4]’, ‘having a tight tummy [P2]’, having heart palpitation and getting ‘a bit shaky [P3]’.

Participants discussed the importance of physical health in terms of the absence of physical health problems. Responses by participants stressed their experience living with comorbidities. One participant's physical health diagnosis led to cascade of issues. A neurological condition affected their balance, resulting in a bone fracture, chronic pain and mental health challenges. Another participant, due to alcohol‐related falls, suffered multiple spinal injuries. These injuries made daily activities more challenging and led to opioid addiction, further complicating chores, shopping and his willingness to try news. ‘It makes me unwilling to try new things. Because, you know, fear of pain, fear of getting hurt, fear of exacerbating the problems, fear of not being able to perform the tasks. I'm just being worried I couldn't do it [P1]’.

Furthermore, the findings showed the influence of illegal substance usage on the motivation levels of the individuals. One participant [P7] provided an example wherein consistent cannabis consumption led to the manifestation of self‐isolation and paranoia. The adoption of self‐isolating behaviour resulted in a decline in motivation and the neglect of physical health. ‘I smoke pot [cannabis] and alternate between smoking pot and getting into an altered state. Until the point that I can't take care of my own needs because I'm too paranoid to go outside [P7]’.

Although people spoke about barriers contributing to their complex physical health experience, they also described enablers leading to improved physical health. They identified that a combination of different health behaviours contributed to improved physical health, including good quality sleep, a healthy diet, physical activity, abstinence from illicit substances and time with family and friends.

P7: Get exercise, eat well, try and stick to a proper sleeping time like sleep schedule.

P2: I guess physical health means getting regular exercise, being active and filling your body with nourishing foods like fruits and vegetables and drinking plenty of water. I went on walks on couple occasions with my friend and her husband. They asked if I want to come for a walk. I walked maybe once or twice a week.

P8: Usually, it's exercising regularly, making sure that I am getting a lot of sleep. For me, that's a really big one. Also spending time with friends and seeing people and taking time for myself.

### 5.2 Role of Medication

The role of medication was a theme that emerged throughout the interview transcripts' analysis. Medication was one contribution to the participants' physical health. In most cases, participants experienced negative side effects of mental health medication. Increased appetite leading to weight gain was part of these side effects.

P2: I've been bigger since taking medication [clozapine].

P3: On this medication [olanzapine], I was eating so much food, especially unhealthy foods. I noticed the changes to my body, how I went from being skinny to gaining weight. After I stopped the medication, I just went back to my normal eating habits with eating vegetables and salads.

Although some participants experienced side effects that negatively impacted their physical health, others reported positive impacts of alternative therapy such as cannabinoid oil. Cannabinoid oil was the perfect pain relief for one participant.

### 5.3 The Importance of the Relationship With Their GP

Participants had mixed GP experiences. Although many valued their GP contact and reported positive interactions, others had negative experiences, prompting them to change GPs or avoid seeking help. Reasons for GP visits varied, from routine check‐ups to mental health concerns. Long waiting lists led some to consult different GPs. Poor communication between GPs and mental health services was a common issue. Negative experiences deterred most from seeing their GP, causing them to consult different GPs. Two participants [P1 and P3] felt marginalised during their GP visit. Some switched to new GPs and now have regular health check‐ups. Those consulting different GPs often had more negative experiences. All participants encountered past negative experiences, driving some to find new GPs for better support.

The analysis revealed key attributes for GPs: listening, understanding, trustworthiness, interest, non‐judgmental attitude and explanations. Trust was vital for feeling taken seriously. One GP exemplified these qualities, creating a comprehensive plan. However, participants felt misunderstood and marginalised when unheard, leading to dissatisfaction. In particular, sedatives were prescribed as a result of one participant's needs not being heard [P3]. Another participant [P8] felt frustrated because their GP did not listen or failed to provide a comprehensive rationale for their actions. Overall, Participants appreciated non‐judgmental, empathic, compassionate approaches, health advice, flexibility and holistic care. They stressed the importance of GPs offering additional resources, like support for housing and food insecurity. Lastly, participants highlighted the need for GPs to have a wider skill set and prioritise holistic care.

## 6 Discussion

This research marks a significant milestone as the first in NZ to explore the perceptions of community mental health service users regarding their physical health and its connection to their experiences with their GP. Previous studies in this field predominantly focused on the perceptions of inpatient mental health service users, often diagnosed with schizophrenia. Notably, an Australian study delved into the relationship between mental health service users and their GPs, revealing the value of a collaborative approach, being heard without judgement and active involvement in decision‐making—an alignment, we find mirrored in our study's findings (Lawn et al. 2021). In this study participants reported concerns of diagnostic overshadowing, feeling labelled and stigmatised and encountering barriers in relation to sedating medication and their complex health issues.

Participants reported not meeting their physical health standards due to weight gain and chronic pain exacerbating their mental health issues. Specifically, the quantitative analysis revealed that 13.8% of participants reported physical health conditions that limited their quality of life within the last 6 months. High blood pressure, asthma, high cholesterol and type 2 diabetes were the most commonly identified diagnoses, indicating that a significant number of people experienced mental illness face chronic health issues that affect their overall wellbeing. The analysis also revealed that individuals who visited their GP more regularly were associated with worse physical health outcomes. Schizophrenia and depression were also found to affect energy levels and physical activity, contributing to weight gain (Vancampfort et al. 2017). Other studies also observed a lack of motivation and energy leading to weight gain in people with mental illness (Happell et al. 2016; Romain et al. 2020). Furthermore, mental health service contact and the use of mental health medication were found to be high among participants. This consistent engagement with mental health services highlight the critical role they play in the lives of people experiencing mental illness.

The Equally Well collaborative in NZ aims to achieve physical health equity for people experience mental health and addiction issues, emphasising the importance of addressing these disparities (Pou 2014). In terms of providing more holistic and integrated options in the community, initiatives such as the Access and Choice programme and the Integrated Primary Mental Health and Addiction Services programme—which includes health improvement practitioners and health coaches located in GP clinic—offer valuable support to individuals seeking comprehensive care (Ministry of Health 2022). These initiative aligns with the participants' aspirations for better health outcomes and reinforces the critical role that health professional play in shaping the perception of individuals experiencing mental illness.

Most participants indicated a need for Kaupapa Māori services, highlighting the importance of culturally competent and safe healthcare practices. Culturally appropriate screening processes that prioritise the unique health needs of Māori individuals are essential to improving health outcomes. Furthermore, Māori‐led solutions, such as community‐based initiatives (e.g., Te Toi Ora ki Whaingaroa) and the involvement of Māori health practitioners, can foster trust and enhance engagement with the health system. By integrating these strategies, we can work towards achieving health equity and ensuring that Māori individuals receive the quality care they deserve.

Accessibility and availability were identified as barriers to equitable physical health care, with free access to GP and gym memberships suggested. Pain and impaired physical function due to pain and weight contributed to poor physical health. Participants felt their weight negatively impacted their overall health, partly due to psychotropic medication. The present study also showed a trend where participants who reported having been informed about potential physical side effects of their medication scored higher on the physical health measure, pointing to the need for better education on the physical consequences of psychotropic medications. In another study, combining medication with health‐promoting strategies helped mental health service users lower their weight (Rollins et al. 2017). The current study also showed that participants who reported a preference for group activities had significantly higher scores for mental health, which indicates a desire for more inclusive and supportive community activities to improve overall wellbeing. This study examines mental health service users' perceptions of their physical and mental health, emphasising their interconnectedness. Participants reported not meeting their physical health standards due to weight gain and chronic pain exacerbating their mental health issues. Schizophrenia and depression were also found to affect energy levels and physical activity, contributing to weight gain. Other studies also observed a lack of motivation and energy leading to weight gain in people with mental illness (Happell et al. 2016; Romain et al. 2020).

The quantitative and qualitative results highlighted that participants strive towards greater physical health. Results indicate that there is an innate desire to experience more agency, become gradually autonomous, and to establish safe and trustworthy relationships with their support person. In general, individuals intrinsically progress towards health (Dunn et al. 2014). This finding contrasts with the belief that mental health service users resist getting healthier (Berger et al. 2013). Nevertheless, the findings of the present investigation challenge these assumptions. Individuals continually strive to optimise their efforts and pursue wellbeing to the best of their abilities, however, they do so within the constraint that many people cannot understand or see. The research participants provided insights into their own views on the concept of health in relation to their own health.

The biomedical model has been widely recognised as the prevailing paradigm in Western medicine, as evidenced by its extensive utilisation in healthcare practises (Stahnisch 2021). This paradigm places its emphasis on the identification and examination of observable signs and symptoms associated with sickness. There is a noticeable paradigm change occurring in NZ and other countries, driven by an increasing body of evidence‐based research that emphasises a holistic approach to individuals' recovery. Today, some approaches in NZ place more emphasis on holistic views that incorporate family and spiritual aspects (Aldersey, Adeponle, and Whitley 2017).

However, some remnants of past health practices influenced by the biomedical model are still apparent. As an example, certain participants adopted a biomedical framework in their perception of health, characterising it as the state of being free from any form of illness or disease. It is evident mental health remains over‐medicalized and the biomedical model, with support from the pharmaceutical companies and psychiatry dictates policies, clinical practice and education around the world (United Nations General Assembly 2015). Health practitioners are still practising according to the biomedical model. Regardless of the international paradigm shift towards a holistic approach, health practitioners have a huge impact on how mental health service users perceive the concept of health and their own health.

The relationship with GPs was essential for participants, emphasising the significance of a good relationship involving listening, support, empowerment, advice and empathy. Diagnostic overshadowing was a barrier, with the physical health of individuals experiencing mental illness often overlooked, especially in primary health care services. Understanding the interconnectedness of physical and mental health is vital for healthcare providers to support individuals experiencing mental illness better. Participants felt stigmatised and labelled due to their mental illness, leading to feelings of worthlessness, self‐isolation, sedentary behaviours and weight gain. Individualised and gradual lifestyle change approaches were found more helpful than sudden adjustments. Implementing a recovery‐oriented approach is essential in primary and secondary health services, but primary health care providers may need familiarity with this approach. Gradual transitions from sedentary to light physical activities can promote physical health in those with mental illness.

## 7 Limitations

Limitations of the study include time constraints of the study timeframe, low response rates and potential biases due to COVID‐19. A cross‐agency approach involving primary and secondary health care services and a holistic and recovery‐focused approach is recommended. GPs should be mindful of diagnostic overshadowing and allow time for meaningful relationships. Collaborative decision‐making and adherence to treatment are crucial.

## 8 Relevance for Practice

All nurses should understand the interconnected relationship between mental and physical health. Nursing education should emphasise this aspect. Mental health professionals can accompany service users to their GP to ensure their concerns are addressed. Multidisciplinary teams can support mental health care delivery. The lack of comprehensive education from mental health professionals highlights the need for in‐depth conversations about possible side effects and holistic well‐being checks. Primary and secondary health staff, such as nurses and doctors, should provide one‐on‐one support with individualised plans, demonstrating empathy for mental and physical pain. Resourceful staff offering individualised plans can best support the physical health of individuals experiencing mental illness. In‐depth conversations and clear communication about tests and diagnoses reduce speculations. Gradual lifestyle changes are more helpful than sudden changes, as they are easier to follow. Exercise practitioners and well‐being coaches can significantly promote healthier lifestyles and reduce stigma among mental health service users.

It is imperative for nurses to cultivate a comprehensive comprehension of individuals' personalised requirements, encompassing their unique preferences, to strive for better health, rather than only prescribing health interventions or imposing dietary choices. Mental health nurses, in particular, should step up in monitoring vital signs and investigating desired health behaviours. Future research could explore peer interviews and evaluate co‐created physical health interventions. In addition, future research could be undertaken to investigate the use of well‐being coaches and exercise practitioners to address mental health service users' comorbidities. Implementing service user experiences across health settings is vital for person‐centred and holistic care.

## Author Contributions

Stefan Sebastian Heinz: conceptualization (lead); writing – original draft (lead); formal analysis (lead); writing – review and editing (equal); methodology (lead); validation (equal); visualisation (lead); investigation (lead). Anthony John O'Brien: supervision (lead); writing – review and editing (equal); validation (equal); conceptualization (supporting); formal analysis (supporting); methodology (supporting); visualisation (supporting). Matthew Parsons: supervision (supporting); validation (equal); conceptualization (supporting); formal analysis (supporting); visualisation (supporting). Cameron Walker: supervision (supporting); validation (equal); formal analysis (supporting); visualisation (supporting).

## Ethics Statement

HREC(Health)2021#35.

## Conflicts of Interest

The authors declare no conflicts of interest.

## Data Availability

The data that support the findings of this study are available from Te Whatu Ora Waikato. Restrictions apply to the availability of these data, which were used under license for this study. Data are available from the author(s) with the permission of Te Whatu Ora Waikato.

## References

[inm13489-bib-0001] Aldersey, H. M. , A. B. Adeponle , and R. Whitley . 2017. “Diverse Approaches to Recovery From Severe Mental Illness.” In The Palgrave Handbook of Sociocultural Perspectives on Global Mental Health, edited by R. G. White , S. Jain , D. M. R. Orr , and U. M. Read , 109–127. London: Palgrave Macmillan, Springer Nature, UK.

[inm13489-bib-0002] Bates, L. , and T. Stickley . 2013. “Confronting Goffman: How Can Mental Health Nurses Effectively Challenge Stigma? A Critical Review of the Literature.” Journal of Psychiatric and Mental Health Nursing 20, no. 7: 569–575. 22906050 10.1111/j.1365-2850.2012.01957.x

[inm13489-bib-0003] Berger, J. L. , M. E. Addis , J. D. Green , C. Mackowiak , and V. Goldberg . 2013. “Men's Reactions to Mental Health Labels, Forms of Help‐Seeking, and Sources of Help‐Seeking Advice.” Psychology of Men & Masculinity 14, no. 4: 433. 10.1037/a0030175.

[inm13489-bib-0004] Cole, M. , and A. Padmanabhan . 2012. “Breast Cancer Treatment of Women With Schizophrenia and Bipolar Disorder From Philadelphia, PA: Lessons Learned and Suggestions for Improvement.” Journal of Cancer Education 27, no. 4: 774–779. 10.1007/s13187-012-0391-7. 22806216

[inm13489-bib-0005] Creswell, J. W. 2011. “Controversies in Mixed Methods Research.” Sage Handbook of Qualitative Research 4, no. 1: 269–284.

[inm13489-bib-0006] Cunningham, R. , D. Peterson , D. Sarfati , J. Stanley , and S. Collings . 2014. “Premature Mortality in Adults Using New Zealand Psychiatric Services.” New Zealand Medical Journal 127, no. 1394: 31–41. 24929569

[inm13489-bib-0007] Curtis, E. , B. Loring , R. Harris , et al. 2022. “Māori Health Priorities. A Report Commissioned by the Interim Māori Health Authority (iMHA) to Inform Development of the Interim New Zealand Health Plan (iNZHP).”

[inm13489-bib-0008] Duggan, M. , B. Harris , W.‐K. Chislett , and R. Calder . 2020. Nowhere Else to Go: Why Australia's Health System Results in People With Mental Illness Getting ‘stuck'in Emergency Departments’, Mitchell Institute Commissioned report 2020, Victoria University.

[inm13489-bib-0009] Dunn, E. C. , A. Winning , N. Zaika , and S. V. Subramanian . 2014. “Does Poor Health Predict Moving, Move Quality, and Desire to Move?: A Study Examining Neighborhood Selection in US Adolescents and Adults.” Health & Place 30: 154–164. 10.1016/j.healthplace.2014.08.007. 25282124 PMC4467831

[inm13489-bib-0010] Durie, M. H. 1985. “A Maori Perspective of Health.” Social Science & Medicine 20, no. 5: 483–486. 10.1016/0277-9536(85)90363-6. 3992288

[inm13489-bib-0011] Every‐Palmer, S. , M. A. Huthwaite , J. L. Elmslie , E. Grant , and S. E. Romans . 2018. “Long‐Term Psychiatric Inpatients' Perspectives on Weight Gain, Body Satisfaction, Diet and Physical Activity: A Mixed Methods Study.” BMC Psychiatry 18, no. 1: 300. 10.1186/s12888-018-1878-5. 30227840 PMC6145113

[inm13489-bib-0012] Frieling, M. A. , W. R. Davis , and G. Chiang . 2013. “The SF‐36v2 and SF‐12v2 Health Surveys in New Zealand: Norms, Scoring Coefficients and Cross‐Country Comparisons.” Australian and New Zealand Journal of Public Health 37, no. 1: 24–31. 10.1111/1753-6405.12006. 23379802

[inm13489-bib-0013] Gray, R. , and E. Brown . 2017. “What Does Mental Health Nursing Contribute to Improving the Physical Health of Service Users With Severe Mental Illness? A Thematic Analysis.” International Journal of Mental Health Nursing 26, no. 1: 32–40. 27982489 10.1111/inm.12296

[inm13489-bib-0014] Happell, B. , S. Ewart , C. Platania‐Phung , J. Bocking , B. Scholz , and R. Stanton . 2016. “What Physical Health Means to Me: Perspectives of People With Mental Illness.” Issues in Mental Health Nursing 37, no. 12: 934–941. 10.1080/01612840.2016.1226999. 27786585

[inm13489-bib-0015] Happell, B. , D. Scott , C. Platania‐Phung , and J. Nankivell . 2012. “Should We or Shouldn't We? Mental Health Nurses' Views on Physical Health Care of Mental Health Consumers.” International Journal of Mental Health Nursing 21, no. 3: 202–210. 10.1111/j.1447-0349.2011.00799.x. 22533327

[inm13489-bib-0016] Hennessy, S. , and A. M. Cocoman . 2018. “What Is the Impact of Targeted Health Education for Mental Health Nurses in the Provision of Physical Health Care? An Integrated Literature Review.” Issues in Mental Health Nursing 39, no. 8: 700–706. 10.1080/01612840.2018.1429509. 29465277

[inm13489-bib-0017] Knaak, S. , E. Mantler , and A. Szeto . 2017. “Mental Illness‐Related Stigma in Healthcare: Barriers to Access and Care and Evidence‐Based Solutions.” Healthcare Management Forum 30, no. 2: 111–116. 10.1177/0840470416679413. 28929889 PMC5347358

[inm13489-bib-0018] Lawn, S. , C. Kaine , J. Stevenson , and J. McMahon . 2021. “Australian Mental Health Consumers' Experiences of Service Engagement and Disengagement: A Descriptive Study.” International Journal of Environmental Research and Public Health 18, no. 19: 181910464. 10.3390/ijerph181910464. PMC8508315 34639765

[inm13489-bib-0019] Ministry of Health . 2020. “Mental Health Data Explorer 2016/17: New Zealand Health Survey.” https://minhealthnz.shinyapps.io/nz‐health‐survey‐2016‐17‐mental‐health‐explorer/.

[inm13489-bib-0020] Ministry of Health . 2022. “Implementation of the Integrated Primary Mental Health and Addiction Services.” https://www.wellbeingsupport.health.nz/assets/IPMHA‐evaluation‐report‐March‐2022‐Malatest‐International.pdf.

[inm13489-bib-0021] Ministry of Health . 2023. Pae Tū: Hauora Māori Strategy. Wellington: Ministry of Health.

[inm13489-bib-0022] Obertová, Z. , N. Scott , C. Brown , A. Stewart , and R. Lawrenson . 2015. “Survival Disparities Between Māori and Non‐Māori Men With Prostate Cancer in New Zealand.” BJU International 115, no. S5: 24–30. 10.1111/bju.12900. 25124231

[inm13489-bib-0023] Plana‐Ripoll, O. , C. B. Pedersen , E. Agerbo , et al. 2019. “A Comprehensive Analysis of Mortality‐Related Health Metrics Associated With Mental Disorders: A Nationwide, Register‐Based Cohort Study.” Lancet 394, no. 10211: 1827–1835. 31668728 10.1016/S0140-6736(19)32316-5

[inm13489-bib-0024] Pou, T. 2014. The physical health of people with a serious mental illness and/or addiction: An evidence review. Auckland: Te Pou.

[inm13489-bib-0025] Robinson, D. J. , M. Coons , H. Haensel , M. Vallis , and J.‐F. Yale . 2018. “Diabetes and Mental Health.” Canadian Journal of Diabetes 42: S130–S141. 10.1016/j.jcjd.2017.10.031. 29650085

[inm13489-bib-0026] Rollins, A. L. , N. H. Henry , A. M. Quash , et al. 2017. “Managing Physical and Mental Health Conditions: Consumer Perspectives on Integrated Care.” Social Work in Mental Health 15, no. 1: 66–79. 10.1080/15332985.2016.1173160. 29308057 PMC5754195

[inm13489-bib-0027] Romain, A. J. , C. Longpré‐Poirier , M. Tannous , and A. Abdel‐Baki . 2020. “Physical Activity for Patients With Severe Mental Illness: Preferences, Barriers and Perceptions of Counselling.” Science & Sports 35, no. 5: 289–299. 10.1016/j.scispo.2020.03.005.

[inm13489-bib-0028] Sinding, C. , L. Watt , P. Miller , et al. 2013. “Stigmas and Silos: Social Workers' Accounts of Care for People With Serious Mental Illness and Cancer.” Social Work in Mental Health 11, no. 3: 288–309. 10.1080/15332985.2012.758075.

[inm13489-bib-0029] Smallwood, R. , C. Woods , T. Power , and K. Usher . 2021. “Understanding the Impact of Historical Trauma due to Colonization on the Health and Well‐Being of Indigenous Young Peoples: A Systematic Scoping Review.” Journal of Transcultural Nursing 32, no. 1: 59–68. 32567510 10.1177/1043659620935955

[inm13489-bib-0030] Stahnisch, F. W. 2021. “Biomedical Dominance, Twentieth Century, and the Establishment of Biomedical Experts.” In Palgrave Encyclopedia of the Health Humanities, 1–9. Cham, Switzerland: Springer.

[inm13489-bib-0031] Te Tiriti o Waitangi . 1840. https://waitangitribunal.govt.nz/en/about/the‐treaty/maori‐and‐english‐versions.

[inm13489-bib-0032] Turner‐Bowker, D. , and S. J. Hogue . 2014. “Short Form 12 Health Survey (SF‐12).” In Encyclopedia of Quality of Life and Well‐Being Research, edited by A. C. Michalos , 5954–5957. Netherlands: Springer. 10.1007/978-94-007-0753-5_2698.

[inm13489-bib-0033] United Nations General Assembly . 2015. “United Nations General Assembly Resolution A.” Antarctica International Law, 15900, 1–35.

[inm13489-bib-0034] Vancampfort, D. , J. Firth , F. B. Schuch , et al. 2017. “Sedentary Behavior and Physical Activity Levels in People With Schizophrenia, Bipolar Disorder and Major Depressive Disorder: A Global Systematic Review and Meta‐Analysis.” World Psychiatry 16, no. 3: 308–315. 10.1002/wps.20458. 28941119 PMC5608847

[inm13489-bib-0035] Ware, J. E. , M. Kosinski , and S. D. Keller . 1996. “A 12‐Item Short‐Form Health Survey: Construction of Scales and Preliminary Tests of Reliability and Validity.” Medical Care 34, no. 3: 220–233. 8628042 10.1097/00005650-199603000-00003

[inm13489-bib-0036] Wells, R. , B. Kite , E. Breckenridge , and T. Sunbury . 2018. “Community Mental Health Center Integrated Care Outcomes.” Psychiatric Quarterly 89, no. 4: 969–982. 10.1007/s11126-018-9594-3. 30090994

[inm13489-bib-0037] WHO . 2022. “Health and Well‐Being.” https://www.who.int/data/gho/data/major‐themes/health‐and‐well‐being.

[inm13489-bib-0038] Wynaden, D. , B. Heslop , K. Heslop , et al. 2016. “The Chasm of Care: Where Does the Mental Health Nursing Responsibility Lie for the Physical Health Care of People With Severe Mental Illness?” International Journal of Mental Health Nursing 25: 516–525. 10.1111/inm.12242. 27416949

